# Zymotic Death-Rates in 1897

**Published:** 1898-03-05

**Authors:** 


### ZYMOTIC DEATH-RATES IN 1897.

The vital statistics for the year 1897 of the 33 large towns dealt with by the Registrar-General show certain changes in the incidence of various zymotic diseases which are worthy of note. Thus the small-pox deaths, which had diminished in the provincial towns, had increased in London. Measles was almost stationary in the provincial towns, although even in them it had diminished a little, viz., from 4,142 deaths in 1896, to 4,120 deaths in 1897. But in London the deaths had gone down enormously in the same time, viz., from 3,697 to 1,929. The deaths from scarlet fever in the provincial towns fell from 1,464 to 1,187, and in London from 942 to 780. Again, in the provincial towns, the deaths from diphtheria fell from 1,519 to 1,156, and in London from 2,683 to 2,261. In whooping-cough, also, the deaths in the provincial towns fell from 3,308 to 2,611, and in London from 2,937 to 1,842. It is the habit of all these diseases to vary in prevalence from year to year, but the length of the oscillation varies with the disease; in the one case a high mortality will occur every alternate year, in another perhaps every third or fourth year; but, curiously, last year there was a diminished mortality from all these diseases at the same time, a diminution which was sufficient to account for a saving of 3,447 lives, as compared with the preceding year, in London alone from these four diseases—measles, scarlet fever, diphtheria, and whooping-cough. Lest, however, we should be too elated by this diminution in one class of zymotic diseases, it should be mentioned that diarrhœa took just the opposite turn, the deaths caused by it having increased between 1896 and 1897 from 3,223 to 4,104 in London, and from 5,537 to 9,513 in the provincial towns.

